# Understanding the Role of Children’s Footwear on Children’s Feet and Gait Development: A Systematic Scoping Review

**DOI:** 10.3390/healthcare11101418

**Published:** 2023-05-13

**Authors:** Yuan Wang, Hanhui Jiang, Lin Yu, Zixiang Gao, Wei Liu, Qichang Mei, Yaodong Gu

**Affiliations:** 1 Faculty of Sports Science, Ningbo University, Ningbo 315211, China; 2 Research Academy of Grand Health, Ningbo University, Ningbo 315211, China; 3 Faculty of Engineering, University of Pannonia, 8200 Veszprém, Hungary; 4 Savaria Institute of Technology, Eötvös Loránd University, 9700 Szombathely, Hungary; 5 Auckland Bioengineering Institute, The University of Auckland, Auckland 1010, New Zealand

**Keywords:** foot health, children’s development, foot disorder, footwear selection, gait development

## Abstract

Children’s footwear plays an important role in the healthy growth of foot and gait development during the growing stage. This review aims to synthesize findings of previous investigations and to explore the biomechanical influences of different types of children’s footwear on foot health and gait development, thus guiding the healthy and safe growth of children’s feet and gait. Online databases were searched for potential eligible articles, including Web of Science, Google Scholar, and PubMed. In total, nineteen articles were identified after searching based on the inclusion requirements. The following five aspects of biomechanical parameters were identified in the literature, including spatiotemporal, kinematics, kinetics, electromyography (EMG), and plantar pressure distribution. Children’s footwear can affect their foot health and gait performance. In addition, children’s shoes with different flexibility and sole hardness have different effects on children’s feet and gait development. Compared to barefoot, the stride length, step length, stride time, and step time were increased, but cadence was decreased with wearing shoes. Furthermore, the support base and toe-off time increased. Double support time and stance time increased, but single support time decreased. The hip, knee, and ankle joints showed increased range of motion in children with the rear-foot strike with larger ground reaction force as well. Future studies may need to evaluate the influence of footwear types on gait performance of children in different age groups. Findings in this study may provide recommendations for suitable footwear types for different ages, achieving the aim of growth and development in a healthy and safe manner.

## 1. Introduction

Children’s shoes can provide surface protection for children’s feet and protect against the wind and rain [1]. This safety protection function enables children to interact fully with the environment and develop their basic motor skills, thus promoting sports participation [2]. Footwear also alleviates the impact of running and encourages children to adopt rear-foot strike mode [3]. It has been shown in studies of children’s shoes to alter lower limb movement, force, and the ability of the foot to perceive stimuli [4], which may contribute to the external forces in the foot–ankle complex during gait [5]. This change from barefoot in primitive humans to shoe-wearing has come a long way.

However, recent studies have shown that habitually barefoot walking children develop well-functioning plantar arches more than their shoe-wearing peers [6,7]. Because the windlass mechanism works more frequently [7], children who are barefoot have more space for the feet and toes to move flexibly. Due to the windlass mechanism, the medial longitudinal arch rises as the contraction of plantar aponeurosis, pulling the calcaneus and extension of the metatarsophalangeal joint during walking and running [8]. Being barefoot has a positive effect in the early stages of life of children whose feet are growing and developing [9]. It was shown that barefoot children spend more time in physical activity each day, which helps to improve foot strength [10]. However, footwear-wearing habits early in life are believed to affect the prevalence of flat feet [11]. Consequently, light and straightforward barefoot shoes have recently gained popularity among parents [12]; the shoes assist children’s foot strength, muscle strength, and balance improvement [13]. Furthermore, the barefoot shoe shows minimal impact and similar motion patterns with being barefoot [8].

Considering the shoe-wearing habit, footwear plays a crucial role in the development of foot and gait in children. Children’s feet have special features, foot shape, and size that can change significantly as they are growing and aging [14]. Compared with adults, there are obvious changes in the form and function of children’s feet [15]. Studies have shown that children’s foot size, ligament strength, and muscle structure change significantly during growth and development [16]. It is worth noting that the development of motor skills is also affected by the functional development of the feet [17]. Foot development involves structural changes in both bone and soft tissue. At birth, the children’s foot is mainly composed of adipose tissue, and ossification gradually begins in the third and fifth prenatal months, followed by the calcaneus, talus, and cuboid [18]. Studies have shown gender differences in the age at the onset and end of scaphoid ossification, with females ranging from 18 months to 2 years and males ranging from 2 to 3.5 years [19]. Muller et al. [20] reported that between the ages of 1 and 6, the foot arch has grown and developed the most rapidly, which showed consistency with Bosch et al. [21]. Muller et al. [20] and Bosch et al. [21] found that at 7 years of age, the arch reached adult levels, and from this age, the arch index becomes stable [22]. Findings also proved that children’s feet grow and develop rapidly between the ages of 12 and 30 months, and in every 2–3 months, children should have new shoes, but the growth rate of feet slowed down significantly as they aged [23]. However, many parents, teachers, and clinicians ignore this and fail to replace children’s shoes in a timely manner.

Wearing uncomfortable shoes with poor fit easily causes flatfoot, hallux valgus, high arch, and other abnormal foot shapes [24,25]. Flatfoot is commonly observed in infants and children, manifested as flexible flatfoot, and usually thought to be the reason that the medial longitudinal arch collapsed [26]. However, there are a few pathological flatfeet conditions, such as congenital vertical talus and tarsal coalition [27]. Causing persistent abnormal pain and skeletal deformity in the foot arch, pathological flatfeet conditions will seriously affect one’s health-related quality of life and should be treated as early as possible. Therefore, research findings on foot growth and development are of great significance for footwear design and manufacture considering the anatomical and physiological characteristics of children [14].

Apart from physiological structure, several factors may contribute to the development of flatfoot in children [15]. Previous studies showed that gender, age, less exercise (sedentary behavior), body weight, degree of joint ligament laxity, ill-fitted footwear, and living environment are contributing factors [27]. As reported, most children in rural regions wore sandals (69.5%), compared to the greater number of children in cities (over 90%) who wore closed shoes [28]. The closed shoes rather than sandals showed greater adverse impact for the longitudinal arch, which may increase the prevalence of flatfoot in urban children compared to rural children [29].

Presented in the children’s cohorts, the hallux valgus is also referred to juvenile bunions, metatarsal varus, or metatarsal adduction, which has complicated pathophysiology and various underlying anatomy. Over 80% of hallux valgus deformities were observed in females, and around half were found at the age of 10 years old [30]. As estimated, around 40–50% of bunions in adults were formed during childhood [31]. The mechanism of hallux valgus is multi-factorial and complex. Age, genetics, flatfoot, varus metatarsal, first metatarsal shape, overactivity, race, and footwear are potential risk factors for hallux valgus. More attention should be paid to the influence of footwear on hallux valgus in children [32]. It was found that Chinese children showed higher ratios of hallux valgus than Mongolian children [33], which may be because the Chinese children wore pointed-toe shoes, which played an important role in contributing to hallux valgus [32]. Previous studies found a substantial connection between hallux valgus angle and shoe fit. Hallux valgus might result from wearing shoes that are either too narrow or too short [34,35], and shorter shoes led to a larger hallux valgus angle [30].

Moreover, studies have reported that people who are habitually barefoot compared to shod people showed a smaller hallux valgus angle [36,37]. This may be because being barefoot may assist in reducing the increased hallux valgus angle resulting from short-length shoes [32]. However, Hollander et al. [9] found different results in all age groups (6–18 years), hallux valgus angles were greater in habitually barefoot adolescents, and the explanation was that the barefoot children enrolled in this study may have had to wear school shoes, which may have been ill-fitting. Therefore, findings of the foot growth and development are of great significance for footwear design and manufacture considering the anatomical and physiological characteristics of children [14]. Therefore, it is necessary to study and discuss different sizes, hardness, and types of footwear for the healthy development of children’s feet to provide inspiration for the clinical evaluation and in-shoe intervention of children’s shoes, to guide the healthy growth of children, and to effectively prevent foot deformities such as flatfoot, hallux valgus, etc.

A few research studies have been conducted on children’s shoes, such as review studies of children’s shoes from Staheli et al. [38] in 1991 and Walther et al. [39] in 2008. However, the two review studies only focused on children’s footwear and did not mention shoe impact on the development of children’s gait and feet. Wegener et al. [5] analyzed the influence of children’s shoes on gait in 2011 and found that footwear affected children’s gait significantly. Children took longer steps and walked faster when wearing children’s shoes. More movement was observed at ankle and knee, and the anterior tibial movement was also greater. Furthermore, the motion of the foot was reduced, while the support phase during gait was increased. Children’s shoes also reduced leg speed during swing of running, reduced foot vibration, and increased the percentage of rear-foot strike patterns [5]. However, since then, many findings from the latest research were not included, and the long-term effect of wearing children’s shoes on gait performance was unknown. Morrison et al. [1] also reviewed the development, biomechanical effect, and clinical treatment of children’s shoes. Cranage et al. [40] in 2019 also reviewed the effects of shoe flexibility on children’s gait. Whilst this review summarized the effects of children’s shoes on time, space, and plantar pressure distribution, it does not report on kinematics and kinetics-related findings.

Therefore, this review aims to synthesize findings of previous investigations and to explore the biomechanical influences of different children’s footwear types on foot health and gait development, thus guiding the healthy and safe growth of children’s feet and gait.

## 2. Materials and Methods

This study followed the PRISMA 2020 Guidelines Reporting project [41] for the checklist employed in the current study.

### 2.1. Inclusion and Exclusion Criteria

Inclusion and exclusion criteria were developed according to the PICO model by PRISMA (population, intervention, comparison, and outcome) to include articles in the systematic scoping review. The inclusion and exclusion criteria of study selection is shown in Table 1.

Inclusion: (1) Studies used shoes as the intervention; research explored elements related to the fit and design of ergonomic footwear; studies looked at how shoes affect a child’s foot growth; investigations examined how footwear affects a child’s stride; research examined how footwear affects a child’s biomechanics; research accounted for published conference proceedings and peer-reviewed publications; and investigations with English-language abstracts available. (2) Healthy toddlers, children, and adolescents; 0–12 years old; sample size > 1. (3) Regular children’s shoes (e.g., sports shoes, ‘Barefoot’ shoes). (4) Research in recent 30 years (1 January 1993–1 January 2023).

Exclusion: (1) Studies did not use footwear as the preliminary or secondary research question; investigations based on commercial design and customization without relation to fit or function; abstracts in non-English language. (2) Unhealthy toddlers, children, and adolescents; greater than 12 years old. (3) Unconventional children’s shoes (e.g., ski boots, skates, therapeutic footwear). (4) Research beyond the 30-year timeline.

### 2.2. Search Strategy

To find eligible studies, the following electronic databases were searched: Web of Science, Google Scholar, and PubMed. The following Boolean search syntax was used: ((Child OR Infant OR children OR toddler OR adolescent) AND (footwear OR shoes) AND (gait OR Plantar Pressure OR Electromyography OR Spatiotemporal OR kinetics OR kinematics)) to search title and/or abstract and/or keywords of articles. Additionally, reference lists of papers as well as conference proceedings and periodicals with biomechanics topics were manually searched.

**Figure 1:**
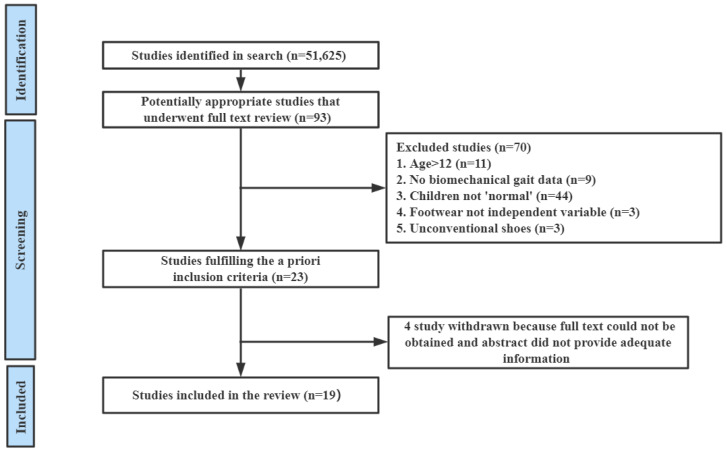
Outline of literature search and inclusion in this study.

Before the literature screening, the duplicate articles were eliminated using referencing software (Endnote) and replenished by manual examination from the principal investigators. The flow diagram of study selection is shown in Figure 1.

## 3. Results

### Search Results

The search results produced 51,625 articles. Finally, 19 studies met the inclusion criteria for this systematic scoping review.

Of the 19 previous studies included, 1 study involved EMG, 7 previous articles studied joint kinematics, and 9 previous articles studied joint kinetics. Similarly, only one article studied plantar pressure. The rest of the article studied the spatiotemporal parameters.

Based on previous research, compared with barefoot running kinematics, footwear led to a reduced hip adduction/abduction and knee flexion/extension, increased ankle angle of motion, and increased incidence of rear-foot strike (RFS) rates [3,9,42,43,44,45,46,47]. In joint kinetics, children running while wearing shoes had greater ground reaction force and lower impact load rate than barefoot running. Running with shoes reduced the axial maximum tibial acceleration, tibial acceleration velocity rate and impact propagation ratio, and increased knee flexion moment and plantar flexion moment [3,48,49]. The influence of soles with different hardness on plantar pressure and EMG of children is different [42]. The gastrocnemius muscles were more active when walking with harder soles [42]. The stiffest soles have the lowest plantar pressure; the softest soft-soled shoes had the highest plantar pressure, similar to barefoot shoes [50]. Shoe-wearing children had longer stride length, step length, stance time, double support time during gait cycle, and wider support base; shoe-wearing children also increased stride time and step time, decreased cadence, and increased walking velocity more than barefoot children [3,8,43,51,52,53,54,55,56].

Specific information on joint kinematics, joint kinetics, and plantar pressure distribution during gait is presented in Table 2 (EMG), Table 3 (Kinematics), Table 4 (Kinetics), and Table 5 (Plantar pressure).

## 4. Discussion

Based on the research results, it was found that children’s footwear can affect their foot health and gait performance. In addition, children’s shoes with different flexibility and sole hardness have different effects on children’s feet and gait development.

### 4.1. Children’s Footwear Effect on Foot

Considering children of different regions and ethnicities, Mauch et al. [57] controlled the factors of sex, BMI, race, and physical activity, and reported that participants in the 6–10 age group who used to go barefoot had feet that were longer and wider. Kusumoto et al. [58] found that the proportion of barefoot children in the Philippines was higher, the feet of Filipino children were shorter than those of Tokyo children, and the foot width and circumference of Filipino children were relatively large. Aibast et al. [10] studied habitually barefoot children of the Kalenjin tribe in Kenya and found that the combination of high levels of physical activity and a barefoot lifestyle resulted in stronger foot muscle strength, ligaments, and tendons. Furthermore, studies comparing children living in the mountains or the Amazon with Ecuadorian children living on the coast have found that coastal Ecuadorian children have longer, wider feet, larger girth, and higher arches [59]. Previous studies reported that children habitually barefoot had longer, wider feet and higher arches than children habitually shod [6,9,10,11,59,60]. Additionally, there was also a link between shoe-wearing age and the incidence of flat feet, with individuals starting shoe-wearing earlier in childhood presenting lower arch heights and higher percentage of flatfoot [61,62,63]. These studies provide more confirmation that regions, ethnicities, climate, and shoe habits will affect children’s foot development [9,10,11,29,57,58,59,60].

Studies found that the barefoot shoes which are light, wide, and flexible may reduce the difference between the forefoot width of walking with shoes and barefoot, thus proposing that moderate minimalist shoes can help children develop foot muscles and improve balance ability and recommending incorporating minimalist footwear features into footwear design and development to facilitate improved child health outcomes [12,13,64,65,66,67].

### 4.2. Children’s Footwear Effect on Gait

Gait patterns of adults showed great changes compared to children, and there are also differences in children’s gait at different ages. Studies found that there are gait changes between children aged 6–7 years and children aged 10–11 years. After 11 years old, gait becomes stable, showing similarity to adults [63]. An updated literature review study may assist in determining the effect of footwear on all facets of children’s gait. This information would facilitate clinical evaluation and in-shoe interventions in pediatric footwear and provide implications for healthy and safe growth for children. The influence of children’s footwear on gait in this review was described in terms of the breadth of biomechanical variables, including spatiotemporal, kinematics, kinetics, electromyography (EMG), and plantar pressure distribution.

Relative to other types of biomechanical variables, the findings of spatiotemporal variables are consistent. Previous research reported that children aged 0–12 years had longer stride length and step length, increased stride time and step time, decreased cadence, wider support base, longer stance time, increased double support time, decreased single support, longer stance time, and increased walking velocity than barefoot children [3,8,43,51,52,53,54,55,56]. Meanwhile, in studies by Heidner et al. [49] and Wolf et al. [8], there was no change in gait velocity between shod and barefoot. Older children (5–11 years) showed increased gait velocity during shod walking compared to barefoot. These spatiotemporal changes in gait may be due to the relative increase in leg length by sole thickness or the increased leg inertia during the swing phase by shoe mass [5]. However, Williams et al. [44] and Gimunová et al. [12] discovered that wearing shoes had no impact on the spatial or temporal parameters.

Footwear with different soles showed different effects on spatiotemporal variables. There has been a debate for many years about whether soft or hard shoes are best for children. Buckland et al. [56] reported that in children who are just beginning to walk, the soft and flexible shoes would reduce step length and stance time during walking. Cranage et al. [51] reported that children wearing hard-soled sandals had shorter stride than those wearing the soft-soled sandals. However, no significant difference in the number of trips and falls when wearing shoes of different hardness was found. Williams et al. [44] found that compared to walking barefoot, soft-soled shoes showed minimal influence on joint kinematics and spatiotemporal parameters measurement in toddlers’ gait. In addition, Cranage et al. [51] and Wolf et al. [8] also discovered that shoes with softer, lighter, and more flexible bottoms had no impact on spatiotemporal characteristics, apart from shorter stride lengths in hard-soled sandals [51]. This is contrary to the findings of Buckland et al. [56] and Williams et al. [44]. The factors that led to this result may be because Wolf et al. [8] did not objectively quantify the changes in footwear stiffness. Still, they were subjectively evaluated, but the more important reason may be due to the differences in children’s age and shoe-wearing time in different experiments. In children who are just starting to walk, the spatiotemporal factors may be affected by unused shoes and less mature gait, individual weight differences of children, and their shoe-wearing history.

There are few studies analyzing the influence of children’s shoes on the plantar pressure distribution. Hillstrom et al. [50] compared the peaks of plantar pressure in children who started walking with four different soles of children’s shoes. The results were significantly different; peak and total pressures in the big toe, first MTP joint, medial and lateral heel, and medial arch were significantly reduced in hard-soled shoes compared with flexible, simple shoes and barefoot conditions. However, the flexible and light shoes had no significant difference in peak pressure in the hallux toe, the first metatarsal joint, the fifth MTP joint, the lateral and medial heel, the medial arch, and overall compared with barefoot shoes [50]. In addition, the stiffest soles have the lowest plantar pressure, which can lead to diminished proprioceptive feedback. Therefore, traditional shoes with stiff soles may not be suitable for children just starting to walk [50]. Conversely, the most flexible soft-soled shoes had the highest plantar pressure, and the pressure division was similar to barefoot conditions [50]. Soft-soled shoes have the least influence on joint kinematics and the spatiotemporal measurement of children’s gait and can best restore the barefoot walking mode [44]. Studies of plantar stress have been limited to children who are just beginning to walk [50]; there is less research on children of other ages, but it is worth noting that BMI may affect the plantar pressure component in children [68]. In obese and overweight children, the prevalence of flat feet is higher than that of normal children, and the interference of BMI and other factors should be strictly controlled in future studies [69] which investigate the effect of shoes on the plantar pressure distribution in the feet of children of different ages.

Several studies found that kinematics of biomechanics could be affected by footwear [3,9,44,45,46,47,49,52]. Compared to barefoot, shoes led to decreased hip adduction/abduction motion, knee flexion, and knee flexion/extension movements, and increased subtalar eversion [44], increased ankle angles compared to barefoot running [3], decreased external rotation of the foot while wearing shoes at midstance and mid-swing [52], and reduced ankle plantarflexion angle at foot strike. Additionally, walking in shoes increased subtalar rotation range of motion (ROM), decreased hallux ROM, forefoot supination ROM, and decreased foot torsion ROM [8]. Wegener et al. [45] reported that shoes decreased midfoot ROM during the contact period in the frontal and transverse planes. Footwear may decrease the rearfoot ROM in the frontal plane, midfoot ROM in the sagittal plane, and transverse plane during propulsion [45]. In addition, the rear-foot strike (RFS) rates are higher when running in shoes [3,49]. Matthias et al. [43] also investigated how shoe size affected the movement of the foot in terms of kinematic parameters. They found that children between ages of 8 and 12 had foot motion frequently restricted with small-sized shoes [43]. Smaller shoes decreased midfoot sagittal plane range-of-motion during walking, thus, in turn, inhibiting hindfoot eversion and first MTPJ dorsiflexion [43].

In addition, the different qualities of hardness and softness of shoes have different kinematic effects. Children had more ankle plantarflexion in barefoot and minimalist conditions as compared to regular running shoes [47]. Hollander et al. [3] also found that different children’s shoes may have different effects on children’s gait, the highest probability of rear-foot strike mode when wearing the cushioned shoe, followed by the minimalistic shoe [3]. Hollander et al. [46] reported that recent studies analyzed the effects of early- and long-term footwear on children’s gait and discovered that younger habitually barefoot children had higher rates of rear-foot strikes during shod and barefoot running and converged in later adolescence. This contrasted with children who regularly used footwear and who grew up using footwear [46]. However, factors such as gender and the hardness of the running surface might influence the results [46].

In terms of kinetics, the walking shoes had smaller vertical ground reaction force than other types of footwear or conditions, such as sports shoes and barefoot, which may be another important distinction between various shoes [48]. Girls showed greater forces values during barefoot than athletic and walking shoes, whilst boys presented higher forces in athletic shoes than barefoot and walking shoes [48].

There are limited numbers of investigations on how children’s footwear affects muscle activities. In the study by Li et al. [42], six 12-year-old female children wore shoes with three different sole hardness, and the intermediate hardness was divided into three different materials. As the findings indicated, the gastrocnemius muscle was more activated during free walking with the harder sole, whereas the tibialis anterior was more activated with the harder sole. In contrast, the lateral femoral and biceps femoris muscles were less affected by the hardness. The tibialis anterior and gastrocnemius muscles, followed by the medial and lateral femoral muscles, are considerably influenced by the hardness of the sole. The tibialis anterior and lateral femoral muscles, as well as the medial and lateral gastrocnemius, are more affected by the sole material [42]. However, this study only investigated female children [14], with a small sample size, and did not investigate male children and children in other age ranges. However, boys and girls start puberty at different times, and the growth and development of lower limb muscles at the same age are also different. Future studies should look at the EMG changes and gender differences associated with shoe-wearing at different ages.

Children’s shoes can affect gait performance; however, whether the influence is essential for functional performance or for long-term foot health and growth is still unknown. There is still a long way to go in understanding the functional effects of footwear and linking theory to practice. Small sample size and failure to control confounding factors still present limitations in the existing literature.

Although this review closely adhered to the inclusion and exclusion criteria, there were still some limitations. Firstly, this paper does not conduct meta-analysis on the obtained studies and data; therefore, the research results are not completely convincing. Secondly, in the previous literature included in the current review study, the sample size varies greatly, and the research hypotheses and analysis methods included in the research are also different, which has a particular impact on the research results. In addition, the human foot is a complex structure, and the development of children’s gait is also a challenging topic. There are many internal factors (gender, age, race, genetics, and BMI) and external factors (footwear, living environment, sports level) that would affect their growth and development. This study has not fully investigated and analyzed related factors. Future work should control for these potential confounding factors and investigate the longitudinal effect of footwear on foot and gait development in children.

## 5. Conclusions

Children’s shoes can affect foot health and gait development. Compared to being barefoot, wearing children’s shoes increases the stance time, stride and step time, and double support time as well as the stride and step length during gait. A decreased cadence, single support, but increased support base, toe-off time was found during gait. The increased range of motion at the hip, knee, and ankle joints may encourage children to use the rear-foot strike mode with higher ground reaction during walking. Children’s shoes with different flexibility and soles with different hardness can affect gait performance.

Therefore, selection of proper footwear in childhood is very important, but a relatively small sample size is still a key problem in current research. Additional research is suggested to assess the effects of various footwear on children’s gaits across different ranges of age groups, to offer clinical implications on the appropriate footwear selection suitable for certain age groups, thus guiding the healthy development and safe growth of children.

## Figures and Tables

**Table 1:** Inclusion and exclusion criteria.

	Inclusion	Exclusion
Research direction	(1) Footwear used as the intervention. (2) Study related to the design and fit of ergonomic footwear. (3) Effect of footwear on children’s foot development. (4) Effect of footwear-wearing on children’s gait and biomechanics was investigated. (5) Research collected from peer-reviewed journals and conference proceedings. (6) Studies with an available abstract in English.	(1) Footwear was not the primary study question. (2) Customization of study designs was based on commercial demand, unrelated to fit or function. (3) Studies without an available abstract in English.
Subjects and age	Healthy infant, children, and adolescents; 0–12 years old.	Unhealthy infant, children, and adolescents; greater than 12 years old.
Footwear	Regular children’s shoes (e.g., sports shoes, ‘Barefoot’ shoes).	Unconventional children’s shoes (e.g., ski-boots, skates, therapeutic footwear).
Retrieval time	Studies in the recent 30 years (e.g., 1 January 1993–1 January 2023).	Studies beyond the 30-year timeline.

**Table 2:** EMG study.

References	Times	Participants	Gender	Nationality of Research Subjects	Age	Shoe Comparison	Result
Cong Li et al. [42]	2018	6	6 girls	China	12–13	Shore A = 50/55/60, Sole material = TPR, MD and Rubber	Higher tibialis anterior activity with SH60, greater gastrocnemius activity with SH55, no difference in biceps femoris and lateral femoral.

**Table 3:** Joint kinematics.

References	Times	Participants	Gender	Nationality of Research Subjects	Age	Shoe Comparison	Result
Hollander, K. et al. [3]	2014	36	22 girls and 14 boys	Australia	6–9	Barefoot shoe, neutral-cushioned running shoe, and minimal shoe	Barefoot run reduced the ankle angle at foot strike. Step length, step width, and rate of rear-foot strike increased.
Matthias, E. et al. [43]	2021	14		Australia	8–12	Bigger, fitted, smaller shoes	Small footwear restricted hindfoot, first MTPJ and midfoot range of motion.
Williams, C. et al. [44]	2021	14		Australia	toddlers	Barefoot and soft-soled shoe (Bobux XPLORER)	Footwear decreased the range of motion of hip adduction/abduction, knee flexion/extension, but increased subtalar eversion.
Wegener, C. et al. [45]	2011	12		Australia	5–13	Barefoot and wearing school shoes	Traditional school shoes restricted children’s foot motion at the midfoot during contact and propulsion phases.
Hollander, K. et al. [9]	2017	678	341 girls and 337 boys	Germany	9–16	Barefoot and shod	Larger hallux valgus angle in all age groups.
Hollander, K. et al. [46]	2018	678	341 girls and 337 boys	Germany	9–16	Barefoot and shod	Higher probability of using rearfoot strikes in habitually barefoot children.
Plesek, J. et al. [47]	2021	48		Czech Republic	3–6	Barefoot/minimalist shoes and standard running shoes	More ankle plantar flexion in the barefoot and minimal shoes.

**Table 4:** Joint kinetics.

References	Times	Participants	Gender	Nationality of Research Subjects	Age	Shoe Comparison	Result
Enrique Alcantara et al. [48]	1996	8	4 girls and 4 boys	Germany	9–11	Unshod vs. shod, casual vs. sport footwear	The rate of load at impact was greater during barefoot running. Shod running reduced maximum tibial acceleration, rate of tibial acceleration, and shock wave transmission. Boys exhibited greater forces in shoes than barefoot, whereas girls had higher values during unshod than in shoes.
Hollander, K. et al. [3]	2014	810	406 girls and 404 boys	Australia	8–16	Barefoot and wearing shoes	Footwear increased maximal and impact ground reaction forces.
Heidner, G.S. et al. [49]	2020	75	G1 = 29 girls; G2 = 16 girls; G3 = 13 boys; G4 = 17 boys	United States	G1 = 4–9; G2 = 3–5; G3 = 6–9; G4 = 4–8	G1 with open toes flat sole, sneakers, and closed toes flat sole; G2 with closed toes flat sole, open toes flat sole, and open toes flat sole; G3 with closed toes flat sole, open toes flat sole, and sneakers; G4 with open toes flat sole, closed toes flat sole, and sneakers. BF for all participants.	No statistical differences in velocity or in vertical and anteroposterior ground reaction force.
Gimunová, M. et al. [12]	2022	30	BF = 8 girls and 7 boys; NBF = 7 girls and 8 boys	Czech Republic	toddlers	BF and NBF	No significant difference.
Moreno-Hernandez, A. et al. [54]	2010	120	59 girls and 61 boys	Mexico	6–13	Barefoot and footwear	The velocity, step and stride length and stance, cadence and swing percentage increased with footwear.
Lythgo, N. et al. [55]	2009	898		Australia	5–13	Barefoot and shod conditions	Gait speed, step length, stride length, support base, step time, stride time, double support stance time increased, but cadence reduced.
Wolf, S. et al. [8]	2008	18	8 girls and 10 boys	Germany	7–9	Barefoot, conventional shoes, and flexible shoes	Stride length and stride time increased, decreased cadence, walking velocity was unchanged with shoes.
Wegener, C. et al. [45]	2011	12	7 girls and 5 boys	Australia	5–13	Barefoot and wearing school shoes	Shoes decreased midfoot range of motion in the frontal and transverse plane during landing. Shoes reduced rearfoot ROM in the frontal plane, midfoot ROM in the sagittal transverse plane during propulsion.
Buckland, M.A. et al. [56]	2014	26	9 girls and 17 boys	United States	toddlers	UltraFlex, MedFlex, LowFlex, and Stiff	Stance time and step width are different.

**Table 5:** Study of plantar pressure distribution.

References	Times	Participants	Gender	Nationality of Research Subjects	Age	Shoe Comparison	Result
Hillstrom, H.J. et al. [50]	2013	26		United States	toddlers	UltraFlex, MedFlex, LowFlex, and Stiff	Stiffest shoe with lowest peak pressures, and the most flexible shoe with the highest pressures.

## Data Availability

The data of literature included for this review may be available upon reasonable request from the corresponding authors.
